# Feeding-related hospitalizations and outcomes in advanced dementia

**DOI:** 10.1007/s41999-025-01366-x

**Published:** 2026-01-28

**Authors:** Ana Rita Ramalho, Maria João Rocha, José Artur Magalhães, Nuno Santos, Isabel Santana, Manuel Teixeira Veríssimo, Lèlita Santos

**Affiliations:** 1 Internal Medicine Department, Hospitais da Universidade de Coimbra, Unidade Local de Saúde de Coimbra, Coimbra, Portugal; 2 Faculty of Medicine, University of Coimbra, Coimbra, Portugal; 3 Neurology Department, Hospitais da Universidade de Coimbra, Unidade Local de Saúde de Coimbra, Coimbra, Portugal

**Keywords:** Advanced dementia, Difficulty eating, Enteral tube feeding, Nasogastric tube feeding, Careful hand feeding, Mortality

## Abstract

**Introduction:**

Dementia is a chronic disease characterized by progressive and mostly irreversible cognitive and functional decline. Advanced dementia is linked to high mortality and morbidity, with over 85% of patients experiencing feeding-related problems. Care should focus on comfort and quality of life, with medical societies recommending against tube feeding, supporting instead careful hand feeding. However, awareness among healthcare professionals regarding tube indications and complications remains limited.

**Purpose:**

To identify the frequency of hospitalizations related to feeding problems, analyze healthcare professionals' attitudes, and compare the outcomes of patients with and without tube feeding.

**Methods:**

This retrospective, observational cohort study analyzed patients aged ≥ 65 years with advanced dementia admitted to Internal Medicine Department of Coimbra's Healthcare Integrated Delivery System between January 1 and March 31, 2023. Sociodemographic, clinical, feeding-related data, along with complications, mortality, and readmissions, were collected and analyzed via STATA.

**Results:**

Of the 1,735 admissions, 178 patients met the inclusion criteria (mean age 86; 64.5% female). Respiratory illnesses were the primary diagnosis (56.7%), and in-hospital mortality rate was 23%. Feeding-related hospitalizations occurred in 17.4% of the patients, mainly due to aspiration. At discharge, 102 patients had no feeding tube (Group A), and 76 did (Group B). Group B had significantly higher complication rates at one year than did Group A, without a survival benefit.

**Conclusion:**

Our findings demonstrate that feeding-related complications also occur in patients receiving enteral tube feeding, without increasing survival or reducing complications and underscore the need for professional training and individualized evidence-based care in advanced dementia.

## Key summary points

**Aim:**

Our study aimed to assess the frequency of hospitalizations related to feeding problems in patients with advanced dementia in an Internal Medicine Department, assess healthcare professionals’ attitudes, and compare outcomes between patients with and without enteral tube feeding.

**Findings:**

We conducted a retrospective cohort study among patients aged ≥65 years with advanced dementia admitted to Internal Medicine Department of Coimbra's Healthcare Integrated Delivery System and found that both feeding-related complications and development of infections are common in the terminal stages of dementia, as 17.4% of admissions were related to feeding problems and 83.1% of the primary diagnosis were infections. We also found that in our cohort, enteral tube feeding did not prolong survival in patients with advanced dementia and did not reduce the incidence of complications.

**Message:**

Feeding-related problems are common in advanced dementia patients and represent a frequent cause of hospitalization in Internal Medicine Departments. Our findings support existing evidence that comfort feeding is a valid and appropriate alternative in this patient population and highlight the need for the early development of an individual and integrated care plan that addresses feeding decisions for individuals with dementia.

## Introduction

Dementia is characterized by significant cognitive decline that interferes with independence [1]. It is a chronic, progressive, and mostly neurodegenerative condition, strongly associated with aging and with a rising incidence [2–4]. It carries high mortality, ranking as the 2nd leading cause of death in high-income countries and is expected to become the 8th cause of disability-adjusted life years by 2050 [2, 3, 5, 6].

Advanced dementia involves severe cognitive and functional impairment, requiring total assistance with daily activities, and is associated with high morbidity and mortality, comparable to late-stage cancer or heart failure [3, 7–10]. Eating problems, affecting over 85% of patients, are related to disease’s progression and also include refusal or forgetting to eat [3, 7, 11–13]. The unpredictable course of dementia often leads to inadequate end-of-life care as survival depends more on care quality than on pharmacological treatment [8, 10, 14, 15]. Comfort and quality of life should therefore be the priorities in end-of-life care, avoiding interventions of questionable benefit [3, 8, 9, 16].

Tube feeding, via nasogastric (NGT), nasojejunal (NJT), percutaneous gastrostomy (PEG), or jejunostomy (PEJ), is one such intervention [9, 17]. Decisions are often influenced by misconceptions about survival or aspiration prevention, limited knowledge of dementia’s course, cultural beliefs, medico-legal issues, and institutional practices [10, 11, 16, 18–23]. However, systematic reviews show no benefit of tube feeding in preventing aspiration pneumonia, improving nutrition, or prolonging life, with some studies reporting lower survival rates [10, 12, 20, 24, 25]. International societies thus discourage tube feeding in end-stage dementia and recommend *comfort feeding*, handfeeding according to patient’s tolerance, focused on comfort rather than nutritional adequacy [7, 9, 11, 23, 26]. Despite this, many professionals remain unaware of the evidence; an Italian study found that over 70% supported artificial nutrition even for end-stage dementia patients [16, 21, 26, 27].

In our country, the Portuguese Society of Internal Medicine (SPMI) and the Portuguese Association for Enteral and Parenteral Nutrition (APNEP) issued a consensus recommending against tube feeding in advanced dementia, endorsing comfort feeding, and recommended individualized care plans defined with patient/legal representative, caregiver, family members, and healthcare professionals, ideally while the patient can still express their preferences [9]. SPMI’s Bioethics Study Group also advised against artificial nutrition when it conflicts with patient values or prolongs suffering without benefit [28].

Given the relevance of this topic and the scarcity of national data, we conducted a 12-month longitudinal, retrospective study of hospitalized patients with advanced dementia to compare outcomes between those with and without tube feeding. Secondary objectives included assessing feeding-related hospitalizations and healthcare professionals’ attitudes.

## Methods

### Study design

We conducted a retrospective longitudinal cohort study utilizing electronic records from patients admitted to the Internal Medicine Department (IMD) of Coimbra Hospital and University Centre (CHUC), Coimbra's Healthcare Integrated Delivery System. Ethical approval was granted by the Ethics Committee of Coimbra’s Healthcare Integrated System (Proc. N°: 2024-ESI-SF.145).

### Participants

Eligible participants were patients aged ≥ 65 years with advanced dementia (Global Deterioration Scale stage 7 [29]), admitted to the IMD between January 1 and March 31, 2023, and followed for one year after discharge. Exclusion criteria included active neoplastic disease, terminal chronic illness, or chronic liver disease (Child–Pugh B or C).

### Data source

Data routinely collected in the electronic records included demographic variables (age, sex, residence).

Feeding decisions before, during, and after hospitalization were analyzed, including prescribed diet type and use of enteral feeding (NGT or PEG). Hospitalization data comprised primary 10th revision of the International Classification of Diseases (ICD-10) diagnoses, admission and discharge dates, reasons for admission, complications (aspiration, restraints, tube dislodgement, pressure ulcers, sialorrhea, and pain/discomfort via the Numeric Pain Rating Scale), and in-hospital mortality. Variables used in the Advanced Dementia Prognostic Tool [30, 31]—dyspnea, pressure ulcers, heart failure, reduced oral intake, weight loss, and body mass index—were gathered to compare post-discharge outcomes between patients with and without tube feeding. Follow-up included 30-day mortality, 30-day readmission, and emergency visits within one year related to feeding problems.

### Statistical analysis

Quantitative variables are presented as means ± standard deviations; qualitative variables as absolute and relative frequencies. Group comparisons used Student’s *t* test for means and proportion tests for categorical data. Differences between means were further assessed through multivariate analysis of variance (MANOVA). A *p* value ≤ 0.05 was considered statistically significant. Analyses were performed using STATA v16 (StataCorp LLC, College Station, TX).

## Results

**Fig. 1:**
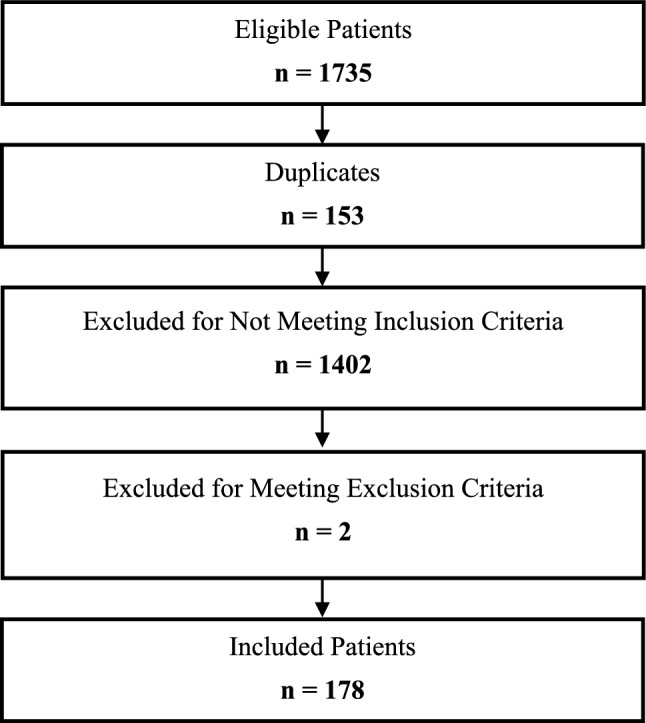
Flowchart of cohort construction

Among 1,735 hospitalizations, 153 (8.8%) were repeated admissions. After excluding these patients, 1,402 patients (88.6%) did not meet the inclusion criteria, and 2 patients (0.1%) met other exclusion criteria—one due to active neoplastic disease and one due to chronic illness in a palliative stage—resulting in a final sample of 178 patients (Fig. 1).

### Patient characteristics

The mean age was 86 years, and most patients were female (*n* = 113; 64.5%) and institutionalized (*n* = 95; 53.4%). Before hospitalization, 129 patients (72.4%) were fed orally, 48 (27%) via NGT, and one (0.6%) via PEG. Diet type was unknown in 52 cases (29.2%) and 6 patients (3.4%) had dietary restrictions. Most NGT-fed patients (*n* = 43; 89.6%) received an enriched liquid diet (Table 1).

**Table 1:** Characteristics of patients with advanced dementia hospitalized in the Internal Medicine Department between January 1 and March 31, 2023 at the Coimbra Hospital and University Centre, Coimbra's Healthcare Integrated Delivery System

Variable	*N* = 178
Age—Mean, in years (SD)	86 (10)
Gender—Female (%)	113 (64.5%)
Modified KATZ Index—*n* (%):	
0—Fully dependent	178 (100%)
Place of origin – n (%):	
Nursing home	95 (53.4%)
Home	70 (39.3%)
National Network of Integrated Continuous Care	13 (7.3%)
Presence of NGT or PEG—*n* (%):	
No	129 (72.4%)
NGT	48 (27%)
PEG	1 (0.6%)
Type of feeding—*n* (%):	
Unknown	52 (29.2%)
Enriched liquid	43 (24.2%)
Creamy	33 (18.5%)
Soft/puréed	24 (13.5%)
Regular	20 (11.2%)
Low-carbohydrate	5 (2.8%)
Low-fat	1 (0.6%)

*SD* standard deviation, *PEG* Percutaneous endoscopic gastrostomy, *NGT* Nasogastric Tube

### Hospitalization

Diet type was mostly enriched liquid diet (*n* = 86; 48.3%) and 14 patients (7.9%) had dietary restrictions.

Infections accounted for 83.1% of primary diagnoses (*n* = 148), and were mainly respiratory (*n* = 95; 53.4%). In-hospital mortality was 23% (*n* = 41). Among survivors, 30-day readmission and mortality rates were 27% (*n* = 37) and 23% (*n* = 32), respectively (Table 2).

**Table 2:** Hospitalization characteristics of patients with advanced dementia admitted to the Internal Medicine Department between January 1 and March 31, 2023 at Coimbra Hospital and University Centre, Coimbra's Healthcare Integrated Delivery System

Variable	*N* = 178
Feeding-related hospitalization—*n* (%)	31 (17.4%)
Type of feeding—*n* (%):	
Enriched Liquid	86 (48.3%)
Creamy	41 (23%)
Soft/Puréed	29 (16.3%)
Regular	8 (4.5%)
Low-Carbohydrate	12 (6.7%)
Low-Fat	2 (1.2%)
TF during hospitalization—*n* (%):	
No prior TF and decision to maintain without TF	73 (41%)
No prior TF and decision to place temporary TF	24 (13.5%)
No prior TF and decision to place TF (maintained at discharge)	39 (21.9%)
With prior TF and decision to maintain	37 (20.8%)
With prior TF and decision to discontinue TF	5 (2.8%)
Primary diagnosis—*n* (%):	
Respiratory infection	95 (53.4%)
Urinary tract infection	22 (12.4%)
Sepsis	14 (7.9%)
Aspiration pneumonia	12 (6.7%)
Heart failure	11 (6.2%)
Infection, unspecified	4 (2.2%)
Dehydration	4 (2.2%)
Acute kidney injury	3 (1.7%)
Respiratory failure	3 (1.7%)
Pulmonary embolism	2 (1.1%)
Skin ulcer	2 (1.1%)
Anal and rectal ulcer	1 (0.6%)
Decompensated diabetes	1 (0.6%)
Gastroenteritis	1 (0.6%)
Paralytic ileus	1 (0.6%)
Neoplasm	1 (0.6%)
Dementia	1 (0.6%)
In-hospital mortality	41 (23%)
30-day readmission	37 (27%*)
30-day mortality	32 (23%*)
Aspiration within 1 year	8 (7.6%**)
Emergency department visits related to feeding within 1 year	9 (8.6%**)
Feeding-related hospitalizations within 1 year	0

TF – tube feeding

^*^Out of a total of 137 patients who did not die during hospitalization

^**^ Out of a total of 105 patients who did not die during hospitalization or within 30 days after discharge

Regarding feeding decisions (Table 3), 41% (*n* = 73) did not initiate tube feeding; 2.8% (*n* = 5) discontinued it; 13.5% (*n* = 24) received it temporarily; 21.9% (*n* = 39) started and maintained it at discharge; and 20.8% (*n* = 37) continued with tube feeding placed prior to hospital admission.

**Table 3:** Description of hospitalizations due to feeding-related issues in patients with advanced dementia admitted to the Internal Medicine Department between January 1 and March 31, 2023 at Coimbra Hospital and University Centre, Coimbra's Healthcare Integrated Delivery System

Variable	*N* = 31		
Age—mean, in years (SD)	86 (5)		
Gender—female (%)	23 (74.2%)		
Place of origin—*n* (%):			
Nursing home	19 (61.3%)		
Home	10 (32.3%)		
National Network of Integrated Continuous Care	2 (6.4%)		
Presence of NGT or PEG—*n* (%):			
No	19 (61.3%)		
NGT	11 (35.5%)		
PEG	1 (3.2%)		
Reason for hospitalization—*n* (%):	$\varnothing$ TF	TF	
Aspiration	6	5	11 (35.5%)
Dehydration	7	2	9 (29%)
Absence of oral intake/Feeding refusal	5	1	6 (19.4%)
Inability to feed via NGT/PEG due to stasis	0	3	3 (9.7%)
Dysphagia	2	0	2 (6.4%)
TF during hospitalization—*n* (%):			
No prior TF and decision to maintain without TF	2 (6.4%)		
No prior TF and decision to place temporary TF	7 (22.6%)		
No prior TF and decision to place TF (maintained at discharge)	12 (38.7%)		
With prior TF and decision to maintain	8 (25.8%)		
With prior TF and decision to discontinue TF	2 (6.5%)		
Complications related to TF—*n* (%):			
None	19 (61.3%)		
Aspiration	6 (19.4%)		
NGT dislodgement	5 (16.1%)		
Physical restraint	4 (12.9%)		
Pressure ulcers	1 (3.2%)		
Pain	1 (3.2%)		
Sialorrhea	1 (3.2%)		
Primary diagnosis—*n* (%):			
Respiratory infection	11 (35.6%)		
Aspiration pneumonia	10 (32.3%)		
Sepsis	3 (9.7%)		
Urinary tract infection	2 (6.4%)		
Dehydration	2 (6.4%)		
Heart failure	1 (3.2%)		
Respiratory failure	1 (3.2%)		
Paralytic ileus	1 (3.2%)		
In-hospital mortality	12 (38.7%)		
30-day readmission	4 (33.3%)		
30-day mortality	7 (58.3%*)		

*NGT* nasogastric tube, *PEG* Percutaneous endoscopic gastrostomy, *SD* standard deviation, *TF* tube feeding

^*^Out of a total of 19 patients who did not die during hospitalization

Feeding problems accounted for 17.4% of hospitalizations (*n* = 31). Patients had a mean age of 86 years, were mostly female (*n* = 23; 74.2%), institutionalized (*n* = 19; 61.3%), and fed orally (*n* = 19; 61.3%). Causes of hospitalization included aspiration (*n* = 11; 35.5%), dehydration (*n* = 9; 29%), refusal or lack of oral intake (*n* = 6; 19.4%), stasis (*n* = 3; 9.7%), and new-onset dysphagia (*n* = 2; 6.4%). Among these patients, the most common decision for those without tube feeding was to initiate tube feeding (*n* = 12; 57.1%). For those already on tube feeding, the most frequent decision was to continue with it (*n* = 8; 80%). Infection was the primary diagnosis in most of these patients (*n* = 26; 84%), predominantly respiratory infections (*n* = 21; 67.9%). In-hospital mortality was 38.7% (*n* = 12). Among survivors, 30-day readmission and mortality were 33.3% (*n* = 4) and 58.3% (*n* = 7), respectively.

### Comparison between patients with and without tube feeding

Table 4 compares patients discharged without tube feeding (Group A, *n* = 102) and with tube feeding (Group B, *n* = 76). Group A included patients for whom the decision was made to maintain oral feeding without tube feeding (*n* = 73), to discontinue tube feeding during hospitalization (*n* = 5), or to use tube feeding temporarily during hospitalization (*n* = 24). Group B included patients for whom the decision was made to initiate tube feeding during hospitalization and maintain it at discharge (*n* = 39), as well as those who were already tube feeding at admission and continued with it (*n* = 37).

**Table 4:** Comparison of postdischarge outcomes between patients with advanced dementia without tube feeding (Group A) and those with tube feeding (Group B) hospitalized in the Internal Medicine Department between January 1 and March 31, 2023 at Coimbra Hospital and University Centre, Coimbra's Healthcare Integrated Delivery System

Variável	Group A *N* = 102	Group B *N* = 76	*p* value
Age—mean, in years (SD)	87 (7)	86 (6)	0.318
Gender—female (%)	63 (61.8%)	50 (65.8%)	0.584
Place of origin—*n* (%):			
Nursing home	51 (50%)	44 (57.9%)	0.296
Home	48 (47%)	22 (29%)	0.015*
National Network of Integrated Continuous Care	3 (2.9%)	10 (13.2%)	0.009*
*The Advanced Dementia Prognostic Tool*—Mean (SD)	15.3 (3.7)	16.4 (3.3)	0.04*
Complications related to TF – n (%):	98 (96.1%)	62 (81.6%)	0.002*
None	0	5 (6.6%)	0.009*
Aspiration	2 (2%)	6 (7.9%)	0.061
NGT dislodgement	1 (1%)	0	0.382
Physical restraint	1 (1%)	7 (7.9%)	0.019*
Pressure ulcers	0	3 (3.9%)	0.044*
Pain	0	3 (3.9%)	0.044*
Sialorrhea	0	1 (1.3%)	0.248
In-hospital mortality	21 (20.6%)	20 (26.4%)	0.369
30-day readmission	19 (18.6%)	18 (23.7%)	0.407
30-day mortality	13 (12.8%)	19 (25%)	0.036*
Aspiration within 1 year	5 (4.9%)	3 (4%)	0.775
Emergency department visits related to feeding within 1 year	7 (6.9%)	2 (2.6%)	0.196
Feeding-related hospitalizations within 1 year	0	0	–

*SD* standard deviation, *NGT* nasogastric tube, *TF* tube feeding

*With statistical significance

Groups did not differ significantly in age (87 vs. 86 years, *p* = 0.318) or sex (61.8% vs. 65.8% female, *p* = 0.584). ADEPT scores differed statistically but not clinically, with similar 6-month mortality predictions (34–43%).

Complications associated with tube feeding were more frequent in Group B (18.4% vs. 3.9%, *p* = 0.002), specifically NGT dislodgement (6.6% vs. 0%, *p* = 0.009), aspiration (7.9% vs. 1%, *p* = 0.019), pressure ulcers (3.9% vs. 0%, *p* = 0.044), and pain (3.9% vs. 0%, *p* = 0.044). No significant differences were observed in in-hospital mortality or 30-day readmission, but 30-day mortality was higher in Group B (25% vs. 12.8%, p = 0.036).

## Discussion

At admission, 27.6% of patients with advanced dementia were receiving enteral nutrition via NGT, a notably high rate given the general decline in NGT use, namely in Europe [23, 32, 33]. Overall 30-day mortality was 41%, aligning with published data [3, 34]. Mortality was higher in patients admitted for feeding-related problems (61.2%), consistent with these marking transition to end-of-life stage [3, 15]. Infections were the main cause of hospitalization, supporting their role as end-stage indicators. Despite this, aggressive treatments with limited benefit and inconsistent with the recommended palliative approach remain frequent, suggesting limited awareness about the natural disease course [3, 13, 23].

Despite the ESPEN recommendation and the SPMI and APNEP consensus statements discouraging tube feeding in terminal dementia, 27.6% of our cohort had NGT at admission, and 21.9% started it during hospitalization, confirming its persistence in practice [7, 9, 21, 23, 35]. Institutional culture and limited training likely influence these decisions. Reinforcing education in geriatrics and palliative care is therefore essential [16, 18, 20, 23, 34, 36]. In our cohort, most tube feeding was administered via NGT, reflecting institutional practices and resource availability that may differ across countries or even among healthcare institutions within Portugal.

Comfort feeding was used in 57.3% of patients. This approach, when supported by family communication, informed discussions, and shared decision-making, particularly through the definition of an individual and integrated care plan, can reduce tube feeding without increasing readmissions [18, 19]. As inability to eat or drink is part of the disease trajectory and hunger is typically absent in terminal stages, hydration should be prioritized and effectively managed by moistening the lips and oral cavity with water or ice chips [3, 7, 13, 37]. The nutritional care approach proposed by *Pessoa* et al. may represent a valuable tool for use in the Portuguese population [9].

While tube feeding in advanced dementia remains a subject of ongoing debate, there is clear evidence that dietary restriction offers no benefit in most patients [7, 9, 11]. Dietary restrictions observed in 3.4% and 7.9% prior and during hospitalization, respectively, only increased the risk of malnutrition [7, 9].

Most patients were institutionalized in nursing homes, supporting ESPEN’s recommendation that these facilities should employ trained staff to assist with feeding and hydration [7]. Training in feeding techniques has been shown to increase the duration of feeding and improve healthcare professionals’ attitudes toward feeding [38]. Training for informal caregivers, as well as financial and social support, are also important and recommended, considering that 39.3% patients lived at home [2, 7].

Our work shows that feeding-related hospitalizations occur even among tube-fed patients. Aspiration was the leading cause of admission and the most frequent complication, reflecting the limited protective role of NGTs. Physiological changes, such as lower esophageal sphincter relaxation, reduced esophageal motility, and poor oral hygiene, may heighten aspiration risk [12, 20, 36]. Dysphagia, although often considered a reason for initiating tube feeding, was the least common feeding-related reason for admission [39]. Most importantly, it may be transient, being frequently triggered by infections, emphasizing the need for formal dysphagia assessment by speech-language therapists or physical and rehabilitation medicine specialists, and consideration of alternatives to address temporary nutritional decline, including dietary supplementation and fortification [7, 39].

When post discharge outcomes were compared, patients with tube feeding showed higher rates of complications and a higher 30-day mortality, indicating that NGT use neither prolongs life nor prevents complications [15, 40, 41]. It is also known that inappropriate use of emergency services is common in patients with dementia [42]. The absence of differences in emergency department visits may be explained by the higher 30-day mortality observed in the tube-fed group [34]. A distinctive feature of our study is the assessment of pain and the development of pressure ulcers [10]. Our findings reinforce international evidence, while creating national evidence, that tube feeding provides no clinical benefit over comfort feeding and highlight the potential advantages of comfort feeding, including reductions in aspiration, sialorrhea, pain, physical restraint, and the development of pressure ulcers [11, 15, 32, 34, 36, 40, 41, 43].

### Implications for clinical practice

Enhancing healthcare professionals’ understanding of dementia thought training in geriatrics and palliative care with focus on the importance of a biopsychosocial approach is crucial to improving quality of life in advanced stages [2, 8, 16, 20, 21, 23]. Early implementation of an individual and integrated care plan—shared with families, aligned with patient preferences, and formally documented in clinical records—that includes directives for managing feeding-related problems should be a priority for professionals dealing with patients with dementia to safeguard their best interests, particularly their comfort and quality of life [3, 7–9, 11, 20, 21, 23, 28, 37].

### Limitations

The main limitation is the retrospective design, which limits causal interpretation and understanding of decision-making factors. The sample size may also explain the numerically small differences, but with statistical significance, observed between the two groups. Although randomized controlled trials would provide stronger evidence, ethical constraints preclude such studies in this vulnerable population [11, 20, 25].

## Conclusion

Feeding-related problems are frequent in advanced dementia and a common cause of hospitalization. Despite clear recommendations advising against tube feeding, its use remains widespread. Our study found that tube feeding neither prevents complications nor prolongs survival, supporting comfort feeding as the preferable alternative. Given the variable clinical course of advanced dementia, there is no one-size-fits-all solution. Early implementation of an individual and integrated care plan addressing feeding decisions is essential to ensure dignity and avoid non-beneficial interventions.
